# Eco-anxiety: What it is and why it matters

**DOI:** 10.3389/fpsyg.2022.981814

**Published:** 2022-09-23

**Authors:** Charlie Kurth, Panu Pihkala

**Affiliations:** ^1^Western Michigan University, Kalamazoo, MI, United States; ^2^Helsinki Collegium for Advanced Studies, University of Helsinki, Helsinki, Finland; ^3^Faculty of Theology, University of Helsinki, Helsinki, Finland; ^4^Helsinki Institute of Sustainability Science, Faculty of Agriculture and Forestry, University of Helsinki, Helsinki, Finland

**Keywords:** wellbeing, eco-anxiety, climate anxiety, moral emotion, emotion, anxiety, climate change, philosophy

## Abstract

Researchers are increasingly trying to understand both the emotions that we experience in response to ecological crises like climate change and the ways in which these emotions might be valuable for our (psychical, psychological, and moral) wellbeing. However, much of the existing work on these issues has been hampered by conceptual and methodological difficulties. As a first step toward addressing these challenges, this review focuses on eco-anxiety. Analyzing a broad range of studies through the use of methods from philosophy, emotion theory, and interdisciplinary environmental studies, the authors show how looking to work on anxiety in general can help researchers build better models of eco-anxiety in particular. The results of this work suggest that the label “eco-anxiety” may be best understood as referring to a family of distinct, but related, ecological emotions. The authors also find that a specific form of eco-anxiety, “practical eco-anxiety,” can be a deeply valuable emotional response to threats like climate change: when experienced at the right time and to the right extent, practical eco-anxiety not only reflects well on one’s moral character but can also help advance individual and planetary wellbeing.

*Greta Thunberg’s activism, borne out of her depression and urge to make a difference, is an example of the positive outcomes climate anxiety can have*. (Borter, 2019)

*Not enough people are experiencing this eco-anxiety*. (Sims et al., 2020)

## Introduction

Increasingly, researchers are investigating the role that emotions play in our response to ecological crises like climate change. This work not only seeks to understand which emotions we experience, but also aims to address evaluative questions about when, if at all, these emotions are useful (for, e.g., individual and planetary wellbeing) and normative questions about whether we ought to experience them. But through much of this work, researchers have only talked in general terms about “climate emotions” or “ecological emotions,” and when they do speak of particular eco-emotions (e.g., “eco-grief,” “eco-anger,” and “eco-anxiety”), they use these labels (and associated empirical measures) in ways that combine different affective responses. So, for instance, we find eco-anxiety conceptualized as a mash up of negative emotions like worry, guilt, and sadness (e.g., Clayton and Karazsia, 2020; Verplanken et al., 2020). We also see discussions of the effects of eco-emotions on pro-environmental attitudes that are grounded in intuitive, but overly-simplistic, emotion models or mere correlational findings (e.g., Kapeller and Jäger, 2020; Stanley et al., 2021).

This is problematic for a variety of reasons. For starters, if we want a better understanding of the nature and value of eco-emotions, we need to recognize (as scholars are starting to do: see, e.g., Ojala et al., 2021) that particular emotions can function in very different ways. And we need to ground our discussions in more sophisticated, empirically-informed accounts of the emotions we are examining. In this paper, we take a step toward developing a more nuanced account of ecological emotions and their value. To do this, we will focus on eco-anxiety.^1^ We take eco-anxiety to be a particularly important case study for several reasons. First, it is increasingly cited as a commonly experienced emotion in the context of ecological crises like climate change. Second, its importance is contested: some think it is valuable, but many do not. Finally, far too little work has been done to understand—both conceptually and empirically—what eco-anxiety is and how it differs from seemingly similar emotions like eco-anger, eco-grief, and eco-guilt (for recent efforts on this, see Ágoston et al., 2022a,b).

Thus, the central aims of this review are three-fold: (i) to identify barriers to, and limitations in, our understanding of the nature and value of eco-emotions like eco-anxiety; (ii) to show how work in philosophy as well as the social and cognitive sciences can help researchers develop better models of eco-anxiety and related emotions; and (iii) to highlight the need for further conceptual and empirical study of ecological emotions and the ways in which they may contribute to individual and planetary wellbeing (for further discussion of planetary wellbeing, see Antó et al., 2021; JYU.Wisdom community, 2021).

Our discussion will proceed as follows. In the section “Existing work on eco-anxiety and the need for a better model of the emotion,” we begin by explaining the need for a better account of eco-anxiety by reviewing a set of conceptual and methodological problems that we find in much of the existing literature on eco-emotions, but that have not yet received sufficient attention. Building from this work, we show that label “eco-anxiety” may be best understood as a term that refers to a *family* of distinct, but related, forms or ecologically-oriented anxiety. In the section “Lessons from emotion science: eco-anxiety as practical anxiety,” we extend this finding by drawing on recent conceptual and empirical research to develop a better account of the form of eco-anxiety that will be our focus. Briefly, what we call “practical eco-anxiety” is the unease that one experiences when thinking about how to respond to ecological threats like climate change. Given the daunting complexity of these situations, one is uncertain about what the best course of actions is; one’s resulting anxiety not only sensitizes one to these challenges, but also prompts the cognitive engagement and motivation that can help one address them.

With this understanding of practical eco-anxiety in hand, we employ methods from philosophy, emotion theory, and interdisciplinary environmental studies in order to argue, in the final two sections, “Discussion” and “Limitations and directions for future research,” that it can be a deeply valuable emotional response to threats like climate change. It contributes, for instance, to our wellbeing, our moral functioning, and the promotion of ecological values. Overall, this review builds on extensive earlier research about eco-anxiety and eco-emotions by the second author: for example, reviews of definitions of eco-anxiety in various disciplines (Pihkala, 2020a), explorations of various climate emotions (Pihkala, 2022), and empirical survey research about young people’s climate anxiety (Hickman et al., 2021).

## Existing work on eco-anxiety and the need for a better model of the emotion

In this section, we discuss three inter-related issues that hamper existing research on eco-anxiety. We suspect that these conceptual and methodological problems result (in part) from the challenges that come when scholars attempt to bring scientific rigor to our everyday talk of eco-emotions. Moreover, while our focus will be on eco-anxiety, the issues we raise infect work on eco-emotions more generally.

### Heterogeneous amalgamations

First consider the tendency to use “eco-anxiety” as the label for a wide range of ecologically-oriented affective experiences. For instance, Verplanken et al. (2020) examine the extent to which “eco-anxiety” correlates with pro-environmental beliefs and behaviors, finding that it does. However, they stipulate that an individual is “anxious” about climate change to the extent that s/he reports high levels of the following seven emotions when thinking about global warming: afraid, nervous, scared, upset, guilty, ashamed, and distressed.

However, given that the emotions on this list have very different functional profiles, this way of defining “anxiety” is problematic. To better see this, notice that if we look at the seven emotions in the Verplanken et al. measure through the lens of standard research on (eco-) emotions (e.g., Scarantino, 2015; Kleres and Wettergen, 2017; Kurth, 2018a; Stanley et al., 2021), it would suggest—not a single affective phenomenon—but rather (at least) three functionally distinct groupings, which might look like this:

- An anxiety-like response (nervous, afraid, and scared): an emotional response to uncertain ecological threats and dangers that engages a broadly defensive response (e.g., risk minimization and risk assessment efforts).
- A self-reflective response (ashamed, guilty): an emotional response concerned with having harmed something of ecological significance that brings tendencies to make amends for the damage done.
- A grief-oriented response (upset, distressed): an emotion focused on the loss of what one sees as ecologically important and that can bring social withdrawal, mourning, etc.

As another example, the Climate Anxiety Scale developed in Clayton and Karazsia (2020) includes many items that seem focused on emotions other than anxiety: for example, “I find myself crying because of climate change” (sadness) and “I feel guilty if I waste energy” (guilt). But in understanding eco-anxiety in such a broad manner, the Climate Anxiety Scale—like the Verplanken et al. measure—appears to conflate distinct affective phenomena (anxiety, sadness, guilt, and anger) that just happen to co-occur with each other.^2^

While measures like these may provide us with insight on our everyday talk about ecologically-oriented “anxiety,” they will cause trouble when used for other purposes. In particular, to the extent that researchers are interested in understanding the role that negative emotions like anxiety, anger, and guilt play in shaping pro-environmental attitudes and behaviors or contributing to (or undermining) our wellbeing, we will need measures that recognize that these emotions are concerned with very different things and so can affect our attitudes and behaviors in very different ways. Without this, we lose the ability to say which emotions are valuable and when. This, in turn, suggests that researchers interested in developing accounts of the nature and value of eco-anxiety will need to look at what empirical research says about what *anxiety*—itself—is and how it functions.^3^

### Overly-simplistic models

Even in work focused on eco-anxiety as a functionally distinct emotion, we find that researchers too often rely on overly-simplistic models or ones that lack sufficient empirical grounding. For instance, eco-anxiety is often presented as a simple, threat-avoidance response: when anxious about ecological matters, we respond to the perceived threat with a stereotyped flight/avoidance response (e.g., Kapeller and Jäger, 2020; Stanley et al., 2021). But it is well-known that anxiety can bring not just flight and avoidance behavior, but also *active engagement* with the perceived danger—things like information gathering efforts, as one seeks to better understand and assess the threat one faces.^4^ Thus this reliance on overly-simplistic models leaves us with an incomplete understanding of how eco-anxiety works. Moreover, in characterizing eco-anxiety as a simple defensive response, these accounts also miss what others have noticed—namely, that eco-anxiety can bring potentially valuable engagement (e.g., Verplanken and Roy, 2013; Lawton, 2019; McQueen, 2021).

There is a similar type of problem in the tendency of some researchers to focus on eco-anxiety’s (near) clinical manifestations. For instance, a 2017 report from the American Psychological Association defines eco-anxiety as the “*chronic* fear of environmental doom” (Clayton et al., 2017, emphasis added). And in the popular media, depictions of eco-anxiety as a paralyzing or pathological state abound (e.g., BBC, 2019; Huizen, 2019).^5^

But while attention grabbing, claims like these need to be interpreted carefully. What these claims typically mean is that there is some *correlation* between experiences of eco-anxiety and self-reports of the *symptoms* of anxiety disorders and depression. Moreover, closer examination of this work also reveals that only a small portion of individuals are experiencing clinical (or non-clinical, but high) levels of eco-anxiety (Berry and Peel, 2018; Clayton and Karazsia, 2020; Ogunbode et al., 2021; Wullenkord et al., 2021).

This is a problem because the tendency to focus on the (near) pathological, but infrequent, instances of eco-anxiety again threatens to distort our understanding of the emotion, its values, and its effects on our wellbeing. To be clear, we are not denying that eco-anxiety can become pathological or undermine a person’s mental or physical life. Rather, the point is that these (near) clinical forms of eco-anxiety are not representative of the emotion as it is typically experienced (Lawton, 2019; Sitra, 2019). In this way, eco-anxiety is not unique: *All* emotions, as features of personality, will exhibit individual differences in how/when they manifest themselves. In a similar vein, one’s life experiences can affect how intensely one experiences a given emotion. For some this will result in a tendency to experience problematic or clinical forms of certain emotions (e.g., anger that gives rise to “explosive anger episodes,” happiness that tends to mania, sadness that brings depression). But for most of us, most of the time, emotional life is not like this. What is needed, then, is a model of eco-anxiety that is oriented, not toward its pathological manifestations, but the phenomenon as a whole.

Related to this is a distinct but similar concern—namely, that claims about how eco-anxiety functions too often lack sufficient empirical backing. For instance, the studies of Stanley et al. are initially presented as investigations of *causal* relationships concerning the “effects of different emotional responses” on (mental) health and behavior (2021: 1). But the studies themselves are *correlational*: they show, for instance, that individuals who report feeling anxious about environmental matters also tend to report experiencing symptoms of depression.^6^ The point here is not that correlational findings are uninteresting, but rather that there is only so much that can learned from them. Without additional work (e.g., intervention studies, structural equation modeling), we cannot say if the emotion causes the behavior or if the behavior is responsible for the emotion—or both. Thus if researchers are to make empirically-grounded claims about what eco-anxiety does, they need more sophisticated models that build from the best emotion science.^7^

### “Eco-anxiety’s” complexity

The project of developing an empirically-grounded account of eco-anxiety is further complicated by ambiguity in the label “eco-anxiety.” It is used both to refer to a unique form of anxiety, and more casually as a term that gestures toward a loosely related—but nonetheless distinct—set of phenomena. To better see this, consider some examples of the very different ways that “eco-anxiety” is discussed in both ordinary conversation and academic research.

First, there is talk of the anxiety that results from considering the enormity of the threat that climate change and the like can bring:

It [eco-anxiety] is something that has always existed in the background, in my thinking about the future and during the majority of my life. … [W]hen you realize how serious, how far-reaching and how fast we need to act in the climate crisis… That’s an experience that shakes your entire foundation, in every possible way. (Berglund, 2019: 33, 27)

I was constantly anxious… I just couldn’t cope… it was an existential crisis… (Hoggett and Randall, 2018: 229; also, Budziszewska and Jonsson, 2021: 12)

Second, there is talk of anxiety in the context of how one’s environmental advocacy will be perceived by others. For instance, Hamilton (2020) discusses the anxiety that some environmental advocates have about coming across as overly moralistic to friends and co-workers (e.g., “I never want to be that [preachy] person”; 155). As another example, Hoggett and Randall (2018) report that some of the deepest anxiety experienced by climate scientists resulted from conflicts with colleagues about whether their work was “giving food to climate deniers” (233).

Finally, there is talk about anxiety in the context of the novel and difficult choices that climate change and other ecological challenges present. Consider, for instance, the following remark revealing an individual’s anxiety about whether to have children:

I am thinking about my future in terms of having kids, and all of that. I don’t know if I really dare to do that, and if that’s kind to have kids in this kind of world. That is something I was thinking about lately, and that’s a really big and scary thought, because I really, really want to have kids, but I don’t feel like it’s safe. (Marczak et al., 2021: 12; also Schneider-Mayerson and Leung, 2020)

Similarly, a recent interview captures how anxieties about environmental dangers can bring heightened awareness and engagement: “I could actually say that environmental anxiety in myself and others drove me to study the subject more. … [My] anxiety worked as a trigger for action” (Pihkala, 2021).

With these quotes in hand, we can now make three observations. First, the themes that these quotes mark out have significant affinities with standard ways that researchers have categorized the various forms of (non-ecological) anxiety that we experience. More specifically, the first set of quotes has the markings of an ecologically-oriented form of existential anxiety.^8^ The quotes of the second set, by contrast, look like social anxiety: the anxieties we feel about the reputational consequences that may come from others’ assessments of us. Finally, the third set provides instances of something that resembles what has been called practical anxiety—the anxiety one feels about what to do in the face of a novel threat or challenge.^9^

Second, though we take these three sets of quotes to be pointing us to distinctive forms of anxiety, we do not see them as all-or-nothing responses. Rather, in the face of a given issue (e.g., whether to have children given climate change), one might experience various forms of eco-anxiety—as well as a range of other emotions. Finally, while each of these three forms of anxiety merits further study, the balance of this review will focus on the third type: eco-anxiety understood as a form of practical anxiety (“practical eco-anxiety,” for short), for it is this form of eco-anxiety that is particularly interesting as a valuable ecological emotion.

## Lessons from emotion science: Eco-anxiety as practical anxiety

In this section, we draw on work in the affective and social sciences to show how it might be used to develop an account of practical eco-anxiety. We begin by fleshing out the idea of practical anxiety as a distinctive form of anxiety in general. We then suggest that some forms of eco-anxiety can be fruitfully understood as a type of practical anxiety. The result will point us toward a new model of eco-anxiety, one that we think is better positioned to provide a detailed, empirically-informed account of eco-anxiety as a distinct emotion.

Before getting started, however, a brief methodological comment is in order. Any effort to look at empirical work on practical eco-anxiety is, as we have noted, complicated by the lack of a common understanding or measure of eco-anxiety in general, much less the particular forms of eco-anxiety introduced above. But these limitations aside, we nonetheless believe that through a close examination of research on eco-emotions, it is possible to identify work that studies eco-anxiety in its various forms (practical, social, existential, etc.), even if labels like “practical anxiety” are not used in much of this work. So while our methodology requires us to be cautious in the conclusions we draw, we believe the results lend support to the account of practical eco-anxiety we are developing.

### What is practical anxiety?

Practical anxiety is the unease that we experience in the face of a novel or difficult choice (Kurth, 2016, 2018a,b,c; Pihkala, 2019). This felt aversiveness serves two purposes. First, it operates as an alarm that helps attune us to the challenges and uncertainty of our situation. Second, it prompts a combination of risk minimization and information gathering efforts aimed at resolving the issues at hand. Moreover, this combination can bring better decisions and the motivation to follow through on them—for the reflection, deliberation, and information gathering that practical anxiety brings is shaped by a concern to get it right, not merely an impulse to rid oneself of the aversive feeling or stave off unpleasant consequences.

To get a more intuitive sense for what practical anxiety is, consider a non-ecologically oriented example: the anxiety you feel about how to care for an aging parent. In such a case, you are anxious about how to, say, reconcile your mother’s medical needs with her long standing wish not to be put in an elder-care facility. Here, your anxiety not only helps you recognize the significance of the decision you face, but also prompts you to get information that will help you enact a better, more informed decision about what to do.

Importantly for present purposes, practical anxiety of this sort contrasts with other forms of anxiety—like the more run-of-the-mill social anxiety you might feel in advance of a blind date or a public talk. With social anxiety, your worries are less focused on *what you should do* (as they are in the case of your mother) and more focused on *how you will be perceived* by others (your date, the audience). Social anxiety, then, is more likely to result in increased caution (even avoidance) on your part as you try to minimize the chance that you will do something that will bring criticism.

In a similar way, this gloss on practical anxiety’s functional role also reveals it to be distinct from superficially similar emotions like guilt and anger. It is not just that these emotions feel different, but that they function in different ways: guilt tends to bring efforts to make up for a wrong done, while anger tends to prompt aggressiveness aimed at redressing an affront to one’s standing.

This intuitive account of practical anxiety (and its contrast with both other emotions and other forms of anxiety) gains empirical backing from work in social and cognitive science. Since these findings are discussed at length elsewhere (see Kurth, 2018a for an overview), we will just give a few examples.

For starters, there is experimental work showing that anxiety about novel or difficult issues brings cognitive engagement (information gathering, reflection, and deliberation) that is aimed at helping one come to a better, more informed decision. One family of studies, for instance, contrasted the behavioral profiles of anxiety and anger. This work found that individuals who experience anxiety in the face of news stories challenging their views on contentious topics (affirmative action, economic policy, and immigration) engage in more—and more open-minded—efforts to learn about the issue. By contrast, those who are angered by the challenging news story tend to seek out less information and primarily look to sources likely to support their pre-existing views (e.g., Valentino et al., 2008; MacKuen et al., 2010; Sweeny and Dooley, 2017).

There is also an extensive set of findings supporting the distinctions between different types of anxiety, including the practical, social, and existential forms of anxiety introduced above. For instance, work in psychology affirms the basic behavioral patterns characteristic of both practical and social anxiety: practical anxiety brings reflection and information gathering efforts in the face of a novel or difficult choice, while social anxiety prompts caution and risk minimization efforts in situations where a negative social evaluation is possible (see, e.g., Leary and Kowalski, 1995; Endler and Kocovski, 2001: chap. 8).^10^

Other research speaks to practical anxiety’s unpleasantness as being central to how it operates. For instance, experimental work shows that anxiety’s tendency to function as an alarm and source of motivation is inhibited when a person is given anti-anxiety drugs or is misled about the nature of their unease (e.g., it is not anxiety, but the effects of the caffeine; Perkins et al., 2013; Jackson et al., 2016).

All told, then, a diverse body of empirical work points to practical anxiety as a functionally distinct form of anxiety: one that is characteristically concerned with questions about what to do, and that brings cognitive engagement and motivation aimed at helping one work through the uncertainty at hand.

### Eco-anxiety as form of practical anxiety

In line with the earlier discussion, it is now easier to appreciate the claim that the form of eco-anxiety captured by the third set of quotes from the section “Eco-anxiety’s complexity” is best characterized as a form of practical anxiety about ecological matters. That is, practical eco-anxiety is the anxiety that one experiences when one is uncertain about what the right thing to do is, and where the practical issue that one is uncertain (and so anxious) about concerns how to respond to ecological threats and challenges. So understood, eco-anxiety, is likely to be elicited by questions such as:

- Should I have a child given the risk that climate change poses to her future?
- Should I change my profession or should I try to bring more environmental responsibility into the job that I have?
- Should I spend more time raising awareness about climate change in my community—and should I do that even if it means spending less time with my family?

Not only does sociological research confirm that people feel eco-anxiety about questions like these (e.g., Berglund, 2019; Schneider-Mayerson and Leung, 2020; Jones and Davison, 2021; Marczak et al., 2021), but it also highlights how these anxieties engage both reflection about what to do and a motivation to act accordingly. This can be seen, for instance, in the earlier quote about whether to become a parent as well as comments like this: “My reaction to that feeling [anxiety] is to just get on and do the next thing… I feel that I’ve got a grip of things, I’m in control.” (Hoggett and Randall, 2018: 237; also: Pearse et al., 2010; Kleres and Wettergen, 2017).

But despite the parallels with the earlier example of your anxiety about how to care for your aging mother, one might worry that these quotes are cherry-picked. So it is worth noting that the account of practical eco-anxiety presented here is grounded in more than just sociological interviews.

First, the idea that eco-anxiety brings responsiveness to environmental threats makes predictions that are supported by research in psychology. More specifically, if eco-anxiety accentuates one’s sensitivity and responsiveness to uncertainty about how best to respond to environmental threats, then there should be a positive correlation between uncertainty about environmental matters and feelings of eco-anxiety. Similarly, there should also be a positive correlation between one’s uncertainty about environmental matters and one’s motivation to engage in environmentally-oriented risk assessment and risk minimization efforts.

Recent investigations of climate change skeptics support these predictions (Jylhä et al., 2016; Haltinner et al., 2021). One study, for instance, surveyed two types of climate change skeptics. The first we can call “deniers”—these are individuals who indicate that they do not believe that climate change is happening or that it is caused by human activities. In contrast to the deniers, the “doubters” expressed uncertainty in response to questions about the reality and causes of climate change. In analyzing participant responses to questions about both the emotions they felt when thinking about climate change and their support for pro-environmental policies, researchers found that, in comparison to deniers, doubters experienced *more worry* and showed *greater support* for pro-environmental policies (Haltinner et al., 2021). Since doubters, but not deniers, are *uncertain* about the reality and human causes of climate change, these results support our predictions about the connection between uncertainty, eco-anxiety, and pro-environmental engagement.

There is also a further set of findings pointing to connections between anxiety and pro-environmental attitudes. The overall theme in this work is that self-reported anxiety about environmental threats correlates with support for pro-environmental policies and actions (e.g., Smith and Leiserowitz, 2014; Albertson and Gadarian, 2015; Chen, 2016; Bouman et al., 2020; Goldberg et al., 2021; c.f., Hart and Nisbet, 2012). Similarly, there are findings showing associations between anxiety about environmental threats like climate change and efforts to become more informed about them (e.g., Yang and Kahlor, 2013; Hmielowski et al., 2019).

While studies of the sort just discussed are important insofar as they point to a connection between anxiety and pro-environmental attitudes/actions, we have also seen (in the section “Overly-simplistic models”) that correlations of this sort are silent on whether there is a *causal* tie between feelings of anxiety and the associated pro-environmental beliefs/behavior. Other work helps fill this gap. For instance, a range of studies indicates that anxiety about environmental matters like climate change (e.g., anxiety reported after, say, reading an article on global warming) increases various dimensions of *cognitive engagement*—deliberation, strategic processing, reflection, etc.—and that this engagement, in turn, leads to pro-environmental attitudes and action.

As one example, when individuals look at advertisements designed to provoke feelings of unease and worry about the environmental dangers of electronic waste, those who reported strong feelings of anxiety in response to the advertisement also tended to report both greater cognitive engagement with the ad (e.g., higher scores for questions like, “This ad really made me think”) and stronger positive attitudes toward the pro-environmental message made in the advertisement (Fernando et al., 2016). Moreover, subsequent structural equation modeling supports these as causal connections: anxiety provoked cognitive engagement and this, in turn, resulted in stronger pro-environmental attitudes (Fernando et al., 2016; also, Meijnders et al., 2001a). Similar experimental work points to feelings of anxiety about (e.g.) pollution and climate change as drivers of things like interest in purchasing environmentally-friendly products and signing petitions supporting pro-environmental legislation (Meijnders et al., 2001b; Nabi et al., 2018).

Finally, there is work supporting the claim that there are different types of eco-anxiety. More specifically, on the account being highlighted here, practical eco-anxiety is felt in the face of novel or difficult questions and brings cognitive engagement that is focused on helping one come to the correct decision about what to do. But as noted in the section “Eco-anxiety’s” complexity, there also seem to be instances of ecologically-oriented anxieties that are prompted by concerns about how ones’ (environmental) actions will be perceived by others and that engage a motivation to act in ways that are socially advantageous. If our distinction in the functional profiles of practical and social eco-anxiety is correct, then there should be empirical work affirming not just the functional role of practical eco-anxiety, but also its social counterpart. And there is.

For instance, a recent set of studies manipulated participants’ beliefs about how their environmental decisions would be viewed by others (e.g., buying green products will have a positive/negative effect on their reputation) in an effort to determine how an individual’s concerns about their reputation affect their eco-behavior. Here researchers found not only that worries about how one’s behavior will be perceived affect how one acts, but that one’s decisions about what to do are geared toward bringing one’s behavior in line with social expectations (Sexton and Sexton, 2014; Brough et al., 2016; Hwang and Choi, 2018). That is, we get results that match our account of social eco-anxiety—anxiety that is driven by motivation to *act in ways that are socially advantageous*. This work then contrasts with the findings discussed above where researchers did not seek to manipulate the participants’ beliefs about how their actions would be perceived by others. Those findings, as we noted, affirm the functional profile of practical eco-anxiety—anxiety shaped by a concern to *make the correct decision*.

### Pay-off: Eco-anxiety as distinct and distinctive

While there is still much that researchers do not know about eco-anxiety, the results discussed here bring conceptual refinement and empirical support to our account of eco-anxiety. As we have seen, there is a wealth of evidence showing that the functional profile of practical anxiety (the general emotion) differs not only from seemingly similar emotions like sadness, anger, and guilt, but also other forms of anxiety (social, existential). Moreover, when we move from genus to species—that is, when we move from practical anxiety in general to practical *eco*-anxiety in particular—we find a growing body of theoretical and empirical support for it as a distinct eco-emotion. The result, then, is an account of practical eco-anxiety that speaks to the conceptual and methodological worries raised above in the section “Existing work on eco-anxiety and the need for a better model of the emotion.”

Before moving on, it is worth pausing to highlight several features of practical eco-anxiety’s functional role—features that draw out not just its distinctiveness as an ecological emotion, but also foreshadow the unique ways in which it can be valuable. First, while eco-anxiety is like other negatively valenced eco-emotions in that the felt aversiveness of these states is central to their functioning, there is an important difference in how eco-anxiety’s alarm-like feeling directs our attention. More specifically, in eco-grief, eco-guilt, and eco-anger we have emotions that are, in the first place, “backward looking” in the sense that they tend to be concerned with things that have already happened (e.g., one is eco-guilty about having taken the trans-Atlantic flight). Eco-anxiety, by contrast, is principally “forward looking” in the sense that it tends to be concerned with decisions or issues that one currently faces or that are on the horizon. Thus, one is eco-anxious about how best to respond to the utility company’s recently announced plans for a new coal-fired power plant or whether one should have children.

But these differences in directions of focus also help draw out how eco-anxiety can be a distinctly valuable emotion. While backward looking eco-emotions are *reactive* responses, eco-anxiety is—in a real sense—a *pro-active* one. It is the emotion that gets one engaged with ecological crises like climate change in the first place: feeling eco-anxious alerts one to the environmental challenges that we face and prompts cognitive engagement that can not only help one think through those challenges, but can also help sustain one’s efforts to address them.^11^

## Discussion

### Eco-anxiety’s importance for agency and wellbeing

Having specified the form of anxiety that we are interested in, we now turn to questions about its value. The basic picture should be apparent from what we have seen so far: eco-anxiety functions as an alarm—it sensitizes individuals to situations where they face a novel or difficult decisions about something of ecological value; and it prompts the cognitive engagement and motivation that can help them address the difficulty they face. Thus, eco-anxiety is an emotion that brings forms of awareness and engagement that can contribute positively to environmental stewardship and agency as well as individual and collective wellbeing.

However, this gloss aside, the bigger picture of eco-anxiety’s value is more complicated. For starters, the very thing that enables eco-anxiety to function as an important signal and source of motivation—its aversiveness—is also thought to be a central driver of trouble: it brings high levels of stress that can lead to intense bouts of anxiety that can undermine our wellbeing and agency. High levels of eco-anxiety can for instance, bring both burnout for those who are engaged by the eco-anxiety they feel as well as eco-apathy in those who try to avoid it. Moreover, empirical findings suggest, contra the picture that emerges from the section “Lessons from emotion science: Eco-anxiety as practical anxiety,” that eco-anxiety might actually frustrate pro-environmental behaviors (e.g., Hart and Nisbet, 2012). The discussion that follows will look at each of these issues more closely. The result will be a cautious optimism that—on the whole—eco-anxiety is instrumentally valuable: it is not some magical elixir, but rather an emotion that, when felt at the right time and in the right way, can make an important difference in how individuals respond to potential environmental threats.

Let us start by looking at how eco-anxiety’s aversiveness can be a source of both good and ill. On the good, eco-anxiety can act both as a signal and source of motivation because it is an unpleasant thing to experience. This can be seen, for example, in the qualitative studies from sociologists. A common finding in this research is that feelings of anxiety about (example) the dangers of climate change are seen as important sources of motivation to get—and stay—involved (Kleres and Wettergen, 2017; Berglund, 2019). For instance, one activist noted how the anxiety she felt sustained her efforts to be more environmentally responsible, even in the face of temptations to revert to old habits:

Yes I have to say that I do [engage more in environmental causes], although not as a political act, but more like, I don’t know, maybe some form of anxiety management, maybe. That I try, that I don’t eat meat and I eat a lot of vegan food and I don’t fly and so on, just because it feels too bad.

(Berglund, 2019: 33)

Something similar can be seen in the words of another activist: “I feel that the climate anxiety lessens by being active, and it is also why I am active. To somehow confront the problem more often makes it feel less stressful” (Berglund, 2019: 34). But the importance of eco-anxiety’s unpleasantness also comes out in the empirical studies discussed above. In that work, *feelings of unease* about environmental threats were implicated as the source of the reflection that individuals engaged in when they were made anxious by advertisements or news clips they viewed.

However, as our own experiences make plain, anxiety about how to respond to ecological crises like climate change can be very unsettling: the threats are just so complex and daunting. In some cases, this stress leads to alienation and hopelessness. In other cases, the desire to avoid the unpleasantness of anxiety can bring disengagement or even the denial of one’s anxiety (Haltinner et al., 2021). Unsurprisingly, these tendencies have been documented in qualitative research:

#### Feeling powerless

“People feel like they cannot do anything. And to be honest, it is not going to really have a massive effect anyway” (O’Neill and Nicholson-Cole, 2009: 371).

#### Overwhelmed

“There is a sense of being alienated from the sort of systems that created the whole issue of climate change, that it is such a big issue and… ‘What does my tiny little self do?’ so there is some anxiety and despair that can come from that. How do…we make changes in the political systems…I mean let alone the planet, the state of the planet just feels overwhelming” (Bell et al., 2021: 9).

#### Hopelessness

“When I read about the insects, that their extinction rate is high, or like—it is going faster than predicted before—it disturbed my concentration… And I was more afraid. More anxiety, more hopelessness” (Marczak et al., 2021).

Moreover, in their assessment of findings from interviews like these, the climate researchers Saffron O’Neill and Sophie Nicholson-Cole maintain that vivid presentations of climate change and other environmental threats are “likely to distance or disengage individuals from climate change, tending to render them feeling helpless and overwhelmed when they try to comprehend their own relationship with the issue” (2009: 375). While the issues here are complicated (a point we return to below), O’Neill and Nicholson-Cole are tapping into a sentiment shared by many climate change researches (Reser and Bradley, 2017).^12^

So, in light of findings like these, researchers should acknowledge that there is a potentially serious down-side to the unpleasantness that eco-anxiety brings. But that said, researchers should *also* keep three things in mind. First, observing that the eco-anxiety of some individuals can come with high levels of stress and hopelessness does not mean that this is the norm (recall the discussion of the section “Overly-simplistic models”). This may be obscured in people’s minds since these milder forms of eco-anxiety are not often called eco-anxiety, and because “eco-anxiety” is a label that is (too) often used to mark out a clinical condition (recall the secction “Existing work on eco-anxiety and the need for a better model of the emotion”).

Second, whether eco-anxiety tends to have these ill-effects will be, in part, a function of a variety of factors. In addition to changes in stress associated with climatic and geophysical change, the eco-anxiety one feels will be shaped by aspects of a person’s social-cultural environment (e.g., the amount of coping resources available in one’s community; see Crandon et al., 2022) and by one’s social position (e.g., being an environmental activist vs. being a climate skeptic), not to mention more specific situational features like one’s coping ability (*cf.* Ágoston et al., 2022a,b). Individual personality differences (i.e., the extent to which one is disposed to be eco-anxious) can also contribute to these dynamics (Helm et al., 2018; Pihkala, 2020a).

Finally, and most importantly, even if eco-anxiety can be too intense in many current circumstances, it does not follow that the *emotion itself* is fundamentally problematic—for research shows that individuals can learn to manage intense, potentially problematic feelings of anxiety. For instance, simple reappraisal strategies like learning to interpret one’s anxiety as a helpful motivator, rather than a debilitating detractor, have been found to be an effective way to moderate worries that would otherwise get the better of us—be it when taking a test (Jamieson et al., 2010), playing sports (Hanin, 2007), or responding to climate change (Ojala, 2012; Grose, 2020; Hamilton, 2020). Similarly, other work suggests that bolstering social support from those one trusts can help one maintain a more healthy level of eco-anxiety (e.g., Hoggett and Randall, 2018).

Thus, looked at from a broader perspective, the conclusion to draw is not that anxiety’s aversiveness tends to be problematic on the whole, but rather (echoing observations from the section “Existing work on eco-anxiety and the need for a better model of the emotion”) that the eco-anxiety that one feels is like any other feature of one’s emotional life: it is something that (with effort) one can learn to use to one’s advantage, and it is something which is strongly shaped by the support (or lack of it) that one gets from one’s surroundings.

Yet skeptics may remain unconvinced: perhaps individuals can get over the unpleasantness that comes with eco-anxiety; but that does not mean that the emotion really works to promote pro-environmental attitudes and behaviors. There are two related issues here: empirical results which suggest that eco-anxiety does little to promote pro-environmental attitudes and actions, and the truism of false-negatives—namely, that individuals often are not eco-anxious when they should be.

Pushing into the details, first consider empirical findings suggesting that anxiety about environmental matters is not helpful. The bulk of this work builds from research on “fear appeals,” that is, advertisements and other communications aimed at arousing fear and anxiety in order to promote precautionary motivations. Here there are studies suggesting that climate-change-focused fear appeals can have a “boomerang effect.” For instance, there are studies suggesting that while ecologically-oriented fear appeals can be effective in promoting pro-environmental attitude among those with liberal political views, they can have the opposite effect with conservatives (Hart and Nisbet, 2012; also, Feinberg and Willer, 2011). Additional research suggests that the effectiveness of these communications is highly sensitive to how the message is delivered (e.g., how severe the environmental threat is presented to be; how much the message suggests one can do to address the threat; Li, 2014). Together, findings like these have led many climate change researchers to conclude that fear appeals are “neither effective nor appropriate in the context of climate change” (Reser and Bradley, 2017: 1).

However, the bigger picture suggests that this pessimistic conclusion is too quick. First, while there is a large body of experimental research examining the effectiveness of fear appeals in general, only a small number of studies have looked specifically at fear appeals focused on climate change and environmental matters. Moreover, meta-analyses of both sets of data point to the same conclusion: well-designed fear appeals can be effective in shaping how individuals think about and approach risky behaviors (e.g., de Hoog et al., 2007; Peters et al., 2013; Reser and Bradley, 2017). In light of this, the core issue seems less about whether fear appeals work, and more about how to design them to promote pro-environmental attitudes and actions. On this front, fear appeals that are sensitive to contexts and which leave individuals with a sense of hope or empowerment look particularly promising (Witte and Allen, 2000; Nabi et al., 2018; Moser, 2019).

Turning to the false-negative problem, that many individuals fail to be eco-anxious is both familiar and well-established (Clayton and Karazsia, 2020; Wullenkord et al., 2021). So if eco-anxiety is valuable, then it seems individuals are not experiencing it often enough. However, granting this, we believe that—here too—the critics’ case for strong skepticism about the value of eco-anxiety is exaggerated. For one, the question of how big the false-negative problem is turns out to be complicated. While it is true that some people just do not seem to experience anxiety about worrisome ecological threats, it is also true that some who may appear to not be eco-anxious actually are—they just do not realize that this is what they are feeling (Haltinner and Sarachandra, 2018; Hoggett, 2019).

But regardless of what the issue is (not feeling eco-anxiety or not realizing one is feeling eco-anxiety), progress is possible. For starters, we have just seen that fear appeals can be effective ways of growing the range of things people are anxious about. Looking beyond fear appeals, other methods for developing a well-tuned disposition to be eco-anxious also show promise. As an example, those with pro-environmental values or a strong environmental identity are more inclined to feel anxious in the face of environmental threats (Clayton and Opotow, 2003; Doherty, 2018; Helm et al., 2018). This suggests that efforts to promote environmental values (e.g., classic environmental education methods such as multi-day opportunities to emerge through nature camps or wilderness outings, see Talebpour et al., 2020) could also help address the false-negative problem, especially if they are integrated with both group- and individual-based methods for coping with difficult emotions (Chawla, 2020; Pihkala, 2020b).

Moving to the second driver, mindfulness training has been shown to improve bodily awareness and so help individuals better identify what they are experiencing (Teper et al., 2013), and thus it does not come as a surprise that some therapists are already advocating for mindfulness as one response to engage with eco-anxiety (e.g., Grose, 2020). Moreover, these improvements may be enhanced through interventions that grow individuals’ emotion vocabulary so that they can better understand—and so control—what they are feeling (e.g., I do not just feel bad, but rather anxious or angry or upset; Lindquist et al., 2006; Davenport, 2017).

Taken as a whole, the findings discussed here provide reason to be cautiously optimistic about our ability to shape eco-anxiety so that it is felt at the right time and in the right way. More generally, the emerging picture suggests that eco-anxiety is, on the whole, an emotion that can make important contributions to our environmental agency and wellbeing.

### Eco-anxiety as a mark of moral attunement

While the above discussion makes a case for eco-anxiety’s value for agency and wellbeing, drawing on insights from moral philosophy suggests that it is importance likely extends further. More specifically, a tendency to feel practical eco-anxiety is not just something that makes individuals more sensitive and responsive to environmental threats. It is also the mark of a morally admirable character. Here Greta Thunberg’s eco-anxiety is a case in point: her anxiety about global warming reveals that she is properly attuned to both what is ecologically valuable and the threats that climate change bring. In this way, her emotions are aligned with what matters in the very way that philosophers dating back to Aristotle take to be the mark of a virtuous individual.^13^

To further draw out this non-instrumental dimension of eco-anxiety’s value, consider some of our earlier examples: the climate change denier, the doubter, and the person thinking about having children. At an intuitive level, the potential parent’s anxiety about whether to start a family because of the threat of climate change (e.g., Schneider-Mayerson and Leung, 2020) shows a *morally admirable sensitivity* to environmental matters that is missing in the denier and deficient in the doubter. Similarly, though both the climate change denier and the doubter are mistaken about the reality and causes of climate change, the doubter’s uncertainty and worries about these issues (e.g., Haltinner et al., 2021) suggest she has a *more admirable ecological attunement*. Moreover, the importance of having one’s emotions properly attuned to environmental matters rests on more than just the fit that these examples have with our everyday intuitions about what is morally admirable; it is also increasingly recognized by political scientists, educational researchers, and psychologists (e.g., Hall, 2021; Ojala et al., 2021; Kurth and Pihkala, forthcoming).

If our reactions to cases like these are on track, it suggests that a disposition to be eco-anxious in the face of potential threats to what is environmentally important is a morally admirable trait. If so, then practical eco-anxiety is both instrumentally *and* non-instrumentally valuable: it is instrumentally valuable insofar as it helps one recognize and response better to morally significant ecological threats, and it is non-instrumentally valuable in that when one’s eco-anxiety is well-tuned, it reflects well on one’s moral character.^14^

Notice as well that, if the model of eco-anxiety that we have been developing is correct, then the practical eco-anxiety that one sees—and admires—in climate activists like Thunberg is not a rarified emotion that only a few exceptional people can experience. Rather, like the emotions that give substance to virtues more generally (Aristotle, 1925; Sherman, 1989; Kurth, 2022: Chap 8), eco-anxiety is an essential ingredient to a morally admirable ecological conscientiousness. Moreover, it—like these other emotions—is a skill-like psychological disposition that (nearly) all of us possess and that (nearly) all of us can shape for the better (Lessons from emotion science: Eco-anxiety as practical anxiety)—as is seen, for instance, in the work of psychotherapists like Dan Rubin whose work has been successful in helping individuals channel emotions like eco-anxiety in productive ways (Wray, 2020; also, Kurth, 2018b). This should leave us optimistic that we can cultivate our eco-anxiety so that we experience it at the right time and in the right way.

## Limitations and directions for future research

This review is one of the first academic texts to integrate philosophical and interdisciplinary inquiry about eco-anxiety. Thus, one limitation is that it is difficult to compare our findings in their totality with other research results.^15^ As a result, the arguments and theoretical framework presented in this review would benefit from future empirical research. There is also a need for further work about both the various dimensions of eco-anxiety (e.g., social aspects and practical aspects) as well as its instrumental value.

One obvious path for future work is to extend this kind of research approach to other eco-emotions, such as guilt and shame. There is emerging scholarship about ecological aspects of these emotions (see Jensen, 2019; Fredericks, 2021; Pihkala, 2022), and that scholarship would benefit from integration with a philosophical and emotion research approach. As with the study of eco-anxiety developed here, some particularly interesting issues for the future study of other eco-emotions like these would include how these negative emotions are related to each other, what contributions they might make to individual and collective wellbeing, and how their benefits might be accentuated.

## Conclusion

By way of conclusion, let us return to the three questions from the beginning of this article: What is eco-anxiety? In what ways might it be valuable? Is it an emotion we ought to feel? Taking these in turn, here is what we can now say. (1) Eco-anxiety can be understood as a form of practical anxiety: an emotion that sensitizes us to difficult decisions about things like climate change, and that brings behaviors (reflection and engagement) aimed at helping us address the difficulty we face. In this way, eco-anxiety is importantly different from other, seemingly similar eco-emotions (e.g., eco-shame, ego-anger, and existential eco-anxiety). (2) The account sketched here, and the empirical support for it, reveal that eco-anxiety can be a valuable emotion, one that helps us see that we face a difficult decision, and one that motivates us to come to a better decision about what to do. While eco-anxiety can also undermine our agency and wellbeing, we have argued that (like many aspects of our emotional life), it is one we can learn to direct in more productive ways. (3) So understood, a well-calibrated tendency to feel eco-anxiety is a mark of a morally admirable sensitivity to ecological crises. Moreover, given that the negative effects of climate change are surely to disproportionately affect the global poor, the benefits that well-tuned eco-anxiety can bring have normative implications—eco-anxiety is an emotion we ought to be feeling to increase planetary wellbeing.

## Author contributions

CK and PP contributed to the conception and design of the study. CK wrote the first draft of the manuscript. PP wrote sections of the manuscript. All authors contributed to the article and approved the submitted version.

## Conflict of interest

The authors declare that the research was conducted in the absence of any commercial or financial relationships that could be construed as a potential conflict of interest.

## Publisher’s note

All claims expressed in this article are solely those of the authors and do not necessarily represent those of their affiliated organizations, or those of the publisher, the editors and the reviewers. Any product that may be evaluated in this article, or claim that may be made by its manufacturer, is not guaranteed or endorsed by the publisher.

## Footnotes

^1^Some scholars use the term "eco-anxiety" principally to refer to *climate change*-oriented anxiety. However, we use it in a broader sense: to refers to anxiety about ecological disaster in general (but where climate anxiety is a significant part of this). We use terms like "eco-emotions" in a similar manner.

^2^Similar concerns apply to Searle and Gow (2010).

^3^In recent studies, scholars have started to recognize the need for more clarity in the scales regarding eco-emotions (e.g., Hogg et al., 2021; Wullenkord et al., 2021). In particular, negative emotions like eco-anxiety should not be thought of as an undifferentiated type of bad feeling, but as functionally distinct affective phenomena (Verlie, 2019; Pihkala, 2020a; Sims et al., 2020; Ojala et al., 2021; Ágoston et al., 2022a,b).

^4^This diversity in how we respond when anxious is seen both in research on anxiety in general (e.g., Blanchard and Blanchard, 2008; Perkins et al., 2009; Kurth, 2018a) and in work on ecologically-oriented anxieties (e.g., Hoggett and Randall, 2018; Kapeller and Jäger, 2020; Lewis et al., 2020; Pihkala, 2020b).

^5^Along these lines, we see a tendency in this research to emphasize the pathological dimensions of eco-anxiety, even among those who recognize its non-pathological elements (Clayton, 2020; Clayton and Karazsia, 2020; Lewis et al., 2020); and while some are starting to back away from this tendency (Albrecht, 2011), others are not (Coffey et al., 2021).

^6^As another example, see O’Neill and Nicholson-Cole (2009) and note 12 below.

^7^See Chapman et al. (2017) and Wullenkord et al. (2021) for related concerns.

^8^Two points. First, here we gloss over the complexity (and puzzles) of talking about existential anxiety—a term that, itself, has a variety of different meanings and that some argue is not even a real form of anxiety. Second, talk of existential anxiety tends to conflate things we should distinguish: intense feelings of anxiety, and feelings of anxiety that are tied up with existential worries (e.g., one’s place in the world). For discussion, see Kurth (2018a) and Pihkala (2019): Ch 3.

^9^To our knowledge, no existing research that seeks to measure and distinguish between the various forms of eco-anxiety highlighted here. However, recent work has flagged the need for such investigations (see, for instance, Pihkala, 2019; Wullenkord et al., 2021; Ágoston et al., 2022a,b). As noted in the Introduction, a central aim of this review is to highlight the need for more conceptual and empirical work on emotions like eco-anxiety.

^10^This picture is also supported by research in abnormal psychology and neuroscience: this work suggests that the behavioral patterns characteristic of these types of anxiety are the upshot of distinctive underlying mechanisms (Kurth, 2016, 2018a: 77–84).

^11^We are not, of course, denying that one can feel, say, anticipatory (eco-) guilt or retrospective (eco-) anxiety; rather, we are pointing to the tendency for certain emotions to look backward while others look forward. For further discussion, see Kurth (2018a): 11.

^12^While we think there is something to O’Neill and Nicholson-Cole’s conclusion, given the observations from the section "Overly-simplistic models," we are concerned about their ability to substantiate *causal* claims like the one in the text from that qualitative research they have conducted.

^13^See, for instance, Aristotle (1925) as well as the contemporary discussion in Sherman (1989), Annas (2011), and Kurth (2018b).

^14^Kurth (2018a,b): Chap 4, provides an extended defense of practical anxiety as instrumentally and non-instrumentally valuable.

^15^Coffey et al. (2021) have made conceptual analysis of eco-anxiety studies, but their approach does not draw from philosophy and emotion research. They also focus on stronger forms of eco-anxiety, not practical eco-anxiety.
